# Structural characterization and *in vivo* pro-tumor properties of a highly conserved matrikine

**DOI:** 10.18632/oncotarget.24894

**Published:** 2018-04-03

**Authors:** Jordan Da Silva, Pedro Lameiras, Abdelilah Beljebbar, Alexandre Berquand, Matthieu Villemin, Laurent Ramont, Sylvain Dukic, Jean-Marc Nuzillard, Michael Molinari, Mathieu Gautier, Sylvie Brassart-Pasco, Bertrand Brassart

**Affiliations:** ^1^ UMR CNRS/URCA 7369 MEDyC, Université de Reims Champagne Ardenne, UFR Médecine, 51095 Reims, France; ^2^ ICMR, CNRS UMR 7312, UFR de Pharmacie, Université de Reims Champagne-Ardenne, 51096 Reims, France; ^3^ Laboratoire de Recherche en Nanosciences, LRN-EA4682, Université de Reims Champagne-Ardenne, 51100 Reims, France; ^4^ CHU de Reims, Laboratoire Central de Biochimie, 51092 Reims, France; ^5^ Laboratoire de Physiologie Cellulaire et Moléculaire, LPCM - EA4667, Université de Picardie Jules Verne, UFR Sciences, F-80039 Amiens, France

**Keywords:** AGVPGLGVG, VGVAPG, elastin, cancer, structure-function

## Abstract

Elastin-derived peptides (EDPs) exert protumor activities by increasing tumor growth, migration and invasion. A number of studies have highlighted the potential of VGVAPG consensus sequence-derived elastin-like polypeptides whose physicochemical properties and biocompatibility are particularly suitable for *in vivo* applications, such as drug delivery and tissue engineering. However, among the EDPs, the influence of elastin-derived nonapeptides (xGxPGxGxG consensus sequence) remains unknown. Here, we show that the AGVPGLGVG elastin peptide (AG-9) present in domain-26 of tropoelastin is more conserved than the VGVAPG elastin peptide (VG-6) from domain-24 in mammals. The results demonstrate that the structural features of AG-9 and VG-6 peptides are similar. CD, NMR and FTIR spectroscopies show that AG-9 and VG-6 present the same conformation, which includes a mixture of random coils and β-turn structures. On the other hand, the supraorganization differs between peptides, as demonstrated by AFM. The VG-6 peptide gathers in spots, whereas the AG-9 peptide aggregates into short amyloid-like fibrils. An *in vivo* study showed that AG-9 peptides promote tumor progression to a greater extent than do VG-6 peptides. These results were confirmed by *in vitro* studies such as 2D and 3D proliferation assays, migration assays, adhesion assays, proteinase secretion studies and pseudotube formation assays to investigate angiogenesis. Our findings suggest the possibility that the AG-9 peptide present in patient sera may dramatically influence cancer progression and could be used in the design of new, innovative antitumor therapies.

## INTRODUCTION

After transformation, tumor cells acquire accelerated proliferative capacity and spread to other locations. This pathological development is influenced by extracellular matrix components. Interactions between tumor cells and the extracellular matrix (ECM) strongly influence tumor development and affect cell growth, division, survival, migration and metastasis [1, 2]. ECM/cell interactions involve cell adhesion to extracellular macromolecules through cell surface receptors and lead to ECM degradation and the release of bioactive ECM macromolecule fragments known as matrikines. Elastin is the major component of elastic fibers; it is particularly abundant in tissues such as arteries and lung but is also present in skin, breast, cartilage and some ligaments. Elastin proteolysis by elastase-type proteinases (metallo, serine and cysteine families) is linked to the genesis of several diseases affecting elastin-rich organs [3, 4]. This degradation is known to unmask cryptic sites within the ECM and to release matrikines, termed elastin-derived peptides (EDPs) [5, 6]. These EDPs display a wide range of biological activities, influencing cell migration [7, 8], differentiation [9], proliferation, chemotaxis [10, 11], survival, tumor progression [12–14], angiogenesis [15], aneurysm formation and atherogenesis [16]. Among all EDPs described in the literature, with the exception of the GRKRK peptide involved in fibroblast adhesion *via* the αVβ3 integrin, two categories of EDPs are described: VGVAPG, VAPG, VGVPG, VGAPG, (VGVAPG)n and PGAIPG with the xGxxPG consensus sequence and AGVPGLGVG, AGVPGFGVG, GLGVGVAPG and GFGVGAGVP with the xGxPGxGxG consensus sequence. Depending on the growth factors present, xGxxPG peptides have been shown to increase skin fibroblast; smooth muscle cell; lymphocyte; and glioblastoma, astrocytoma, melanoma and lung carcinoma cell proliferation. EDP-dependent cell proliferation is mediated through VGVAPG-derived peptides; this sequence is repeated several times in tropoelastin, which binds to an elastin receptor with lectin-like properties known as elastin receptor complex (ERC) (S-galactosidase/neuraminidase-1/cathepsin A protective protein). A unique sequence in S-Gal (encoded by the frame-shifted exon 5 of β-galactosidase) is believed to be responsible for the cellular response to xGxxPG peptides. S-Gal-mediated signaling is blocked by the antagonist lactose [17]. Lactose-preincubated cells are insensitive to EDP-stimulation, resulting in a lack of cell proliferation.

The xGxPGxGxG peptides have been shown to increase skin fibroblast, endothelial cell and macrophage chemotaxis as well as carcinoma cell adhesion [10, 18–20]. Our previous data showed that xGxPGxGxG peptides increased proteinase release by carcinoma and fibrosarcoma cells [20, 21]. Currently, the receptors involved in xGxPGxGxG peptide-mediated regulation remain unknown. Furthermore, no structural characterization or complete biological property studies have been completed. Towards this aim, we proposed to study xGxxPG and xGxPGxGxG peptides in parallel to determine their biophysical properties and their *in vivo* and *in vitro* protumor capacities.

## RESULTS

### Elastin domain-26 is more conserved than elastin domain-24

**Figure 1:**
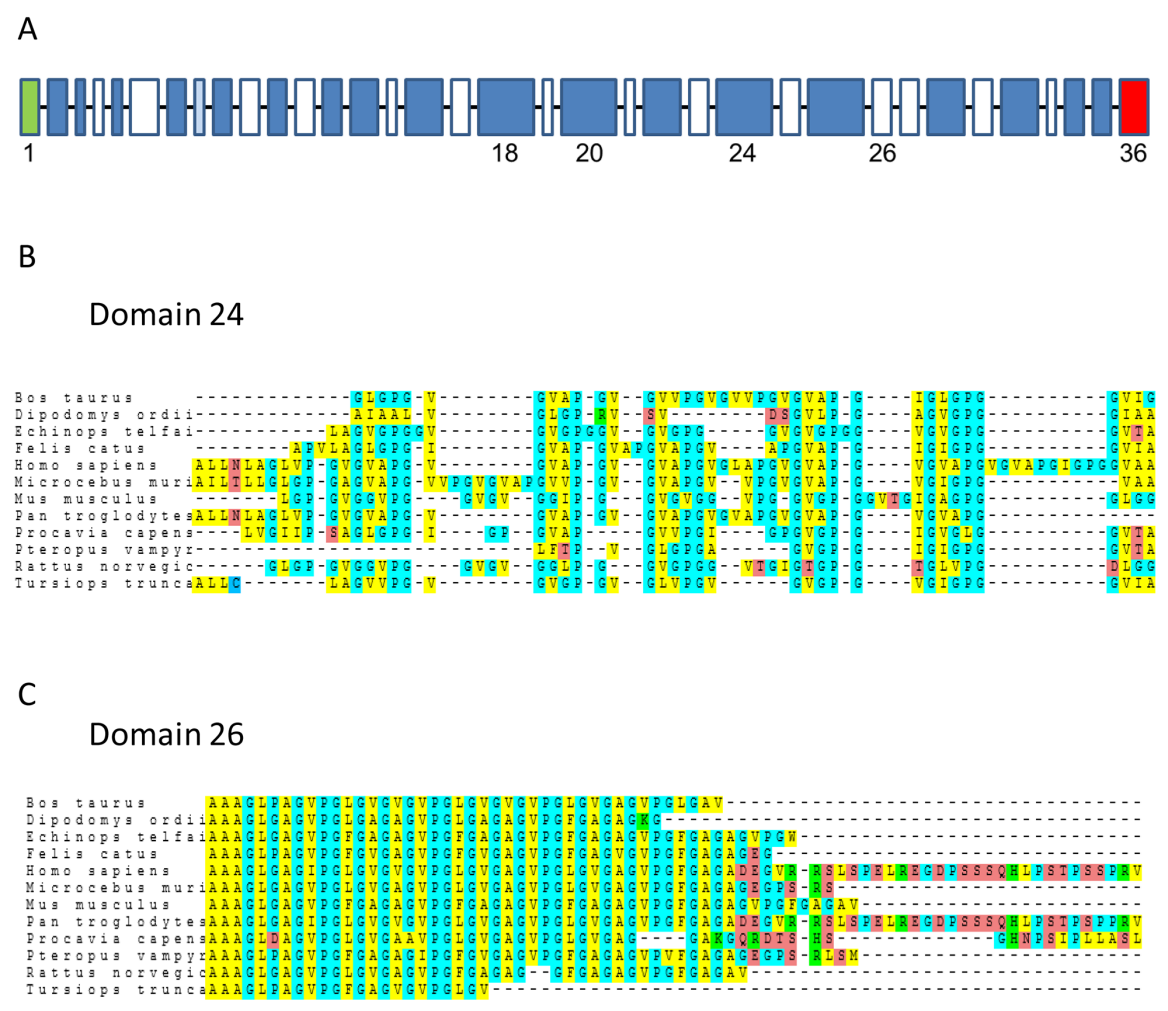
Alignments of domain-24 and domain-26 elastin peptides **(A)** Schematic of human tropoelastin primary organization. All domains are shown. Green domain: signal peptide; blue domain: hydrophobic domain; white domain: cross-linking domain; red domain: C-terminal domain. **(B)** Multi-species alignment of domain-24 elastin peptide from 12 mammalian species. **(C)** Multi-species alignment of domain-26 elastin peptide from 12 mammalian species. Amino acids with similar chemical characteristics are color-coded. The 12 mammalian sequences are derived from Ensembl sequences (Supplementary Table 1).

The elastin gene is located on chromosome 7q11.2 in humans. Synteny in this region is generally well conserved among mammalian species. The style of sequence found in the elastin gene is highly conserved among elastins of different species. The elastin genes consist of exonic sequences that code for long, hydrophobic protein regions interspersed by exonic sequences coding for shorter cross-linking regions (Figure 1A). The sequences of elastin domain-24, which contains the VG-6 peptide, and elastin domain-26, which contains the AG-9 peptide, were analyzed in different mammalian species. Alignment studies showed that elastin domain-26 is more conserved than elastin domain-24 (Figure 1B–1C). For example, the percentage homologies between human and mouse domain-24 and domain-26 were 46.77% and 64.15%, respectively.

### CD spectroscopy

**Figure 2:**
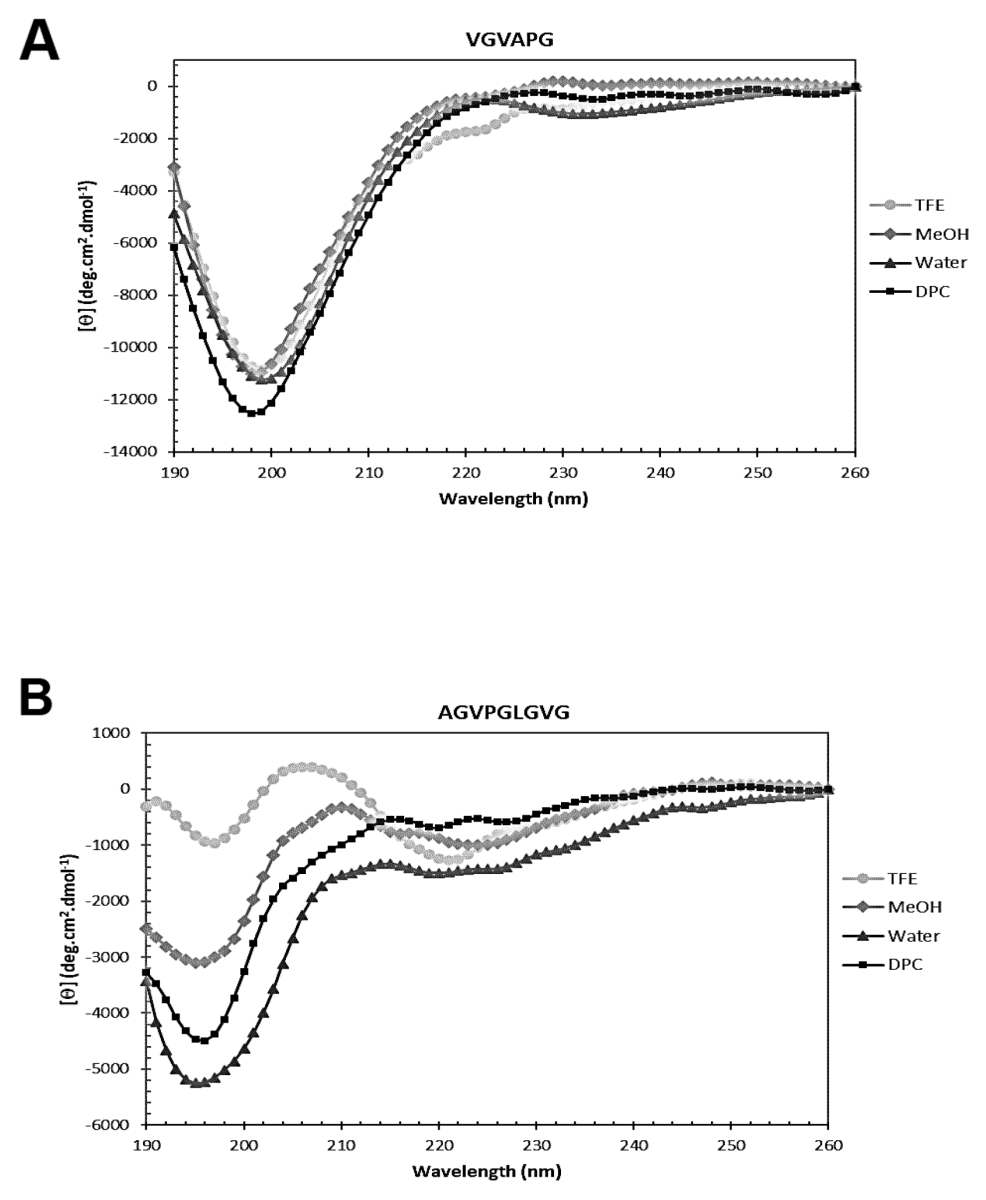
Secondary structure analysis by circular dichroism Far-UV CD spectra of **(A)** VGVAPG (VG-6) and **(B)** AGVPGLGVG (AG-9) dissolved in 30% TFE, 30% MeOH, water and DPC micelles. Spectra were recorded at 293 K using a peptide concentration of 0.1 mM.

Circular dichroism analysis of VG-6 and AG-9 was carried out to assess secondary structures in solution. Figure 2 shows the CD spectra of the VG-6 and AG-9 peptides recorded in H_2_O, H_2_O/TFE (7:3 v/v), H_2_O/methanol (7:3 v/v) and 80 mM DPC-*d*_*38*_ detergents at 293 K.

The VG-6 peptide CD spectra (Figure 2A) are characterized by a main negative dichroic band below 200 nm and a shoulder near 220 nm in both media. This intense negative band likely corresponds to the presence of random-coil or disordered structures, as fully disordered polypeptides are considered to have a negative band near 195 nm [22, 23]. The shoulder at approximately 220 nm that is only slightly more intense in H_2_O/TFE (7:3 v/v) indicates some order, probably corresponding to β-turn conformations of VG-6 [23–25]. However, the intense negative band near 195 nm and the small shoulder at approximately 220 nm may be assigned to PPII conformations, even if the expected positive band at 218 nm is absent [26].

The CD spectra of AG-9 peptide (Figure 2B) dissolved in aqueous and micellar media also displayed an intense negative dichroic band below 200 nm, presumably due to random-coil or disordered conformations. However, adding an organic solvent such as MeOH or TFE to water results in the partial conversion from a less-ordered state to β-turn structures, yielding a mixture of different types of β-turns and unordered conformations [22, 23, 25, 27, 28]. In particular, in H_2_O/TFE (7:3 v/v), the CD spectrum is characterized by a slight negative dichroic band at 197 nm and two additional bands of opposite signs, one positive band at 206 nm and another negative band at 221 nm. This probably indicates the presence of two preponderant types of ordered conformations and suggests the coexistence of different β-turns mixed with random coil conformations [22, 23, 27, 28].

Our CD results highlight that VG-6 and AG-9 dissolved in previous media probably display a mixture of random coil and β-turn structures, which is in accordance with secondary structure studies of other elastin-like peptides published in the literature [29].

### NMR spectroscopy

Conformational analysis of the VG-6 and AG-9 peptides was performed in extracellular-mimetic and membrane-mimetic environments, respectively, in water and in zwitterionic DPC-d_38_ media [30]. The use of a micelle system in liquid-state NMR studies is widespread to mimic water-membrane interfaces due to the high-quality spectra frequently obtained for the solubilized peptides of interest [31]. Although micelles do not form bilayers, they are considered to be pertinent models for characterizing surface-bound peptide conformations and probing the initial states of interaction with biological membrane-embedding receptors [32]. According to the Wüthrich strategy [33], chemical shift deviations (CSDs), ^3^J_NH–Hα_ coupling constants, amide proton temperature coefficients and ROE connectivities have been investigated.

**Figure 3:**
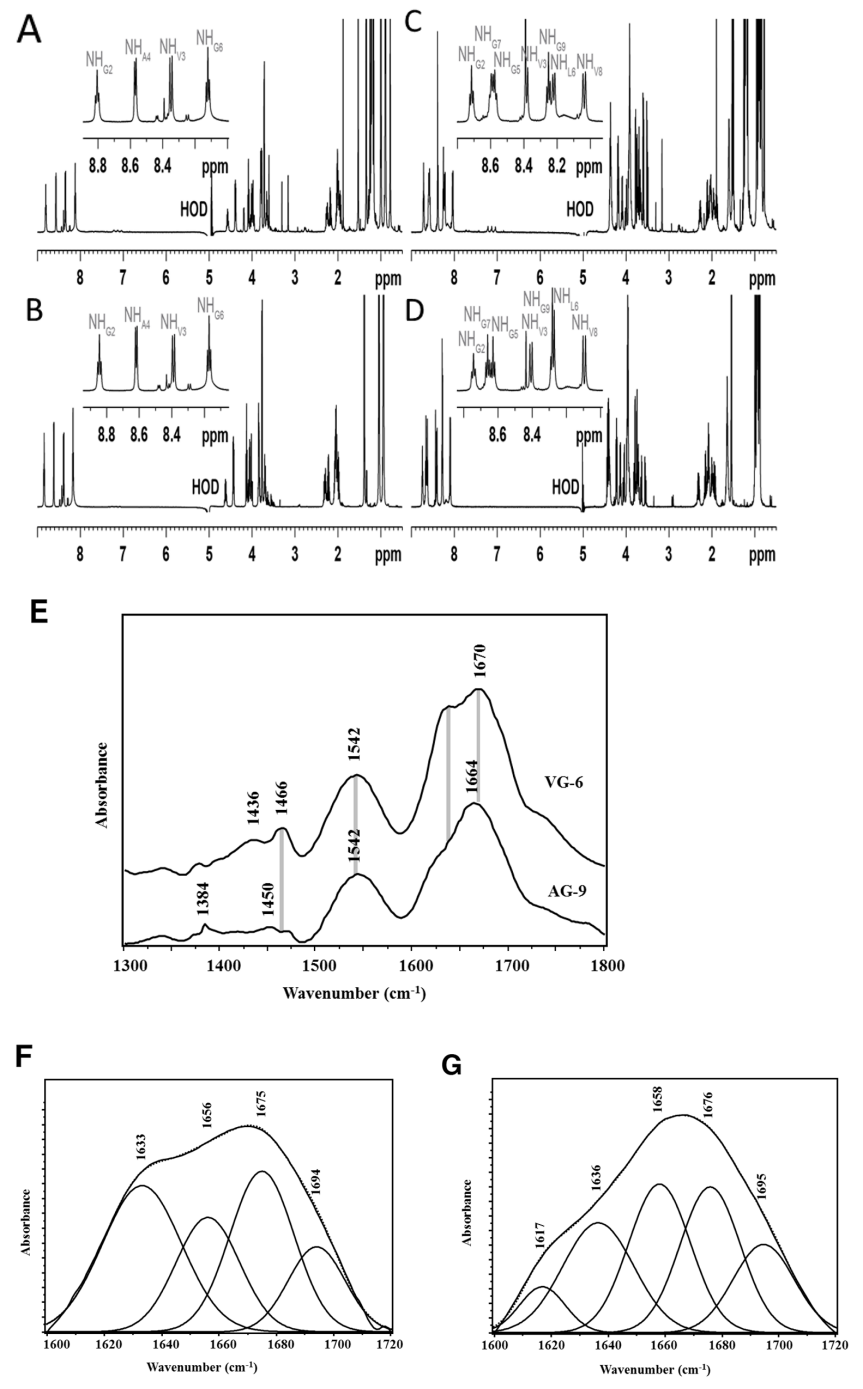
VG-6 and AG-9 1H NMR spectra and secondary structure analysis by FTIR spectroscopy 1D ^1^H spectra of VGVAPG, at 278 K, at 600 MHz (^1^H), dissolved **(A)** in H_2_O/D_2_O (9:1 v/v), at 5 mM, at pH = 5.0 and **(B)** in H_2_O/D_2_O (9:1, v/v) including DPC-*d*_*38*_ micelles (160 mM), at 2 mM, pH = 5.0. 1D ^1^H spectra of AGVPGLGVG, at 278 K, at 600 MHz (^1^H), dissolved **(C)** in H_2_O/D_2_O (9:1 v/v), at 7.5 mM, pH = 5.0 and **(D)** in H_2_O/D_2_O (9:1 v/v) including DPC-*d*_*38*_ micelles (160 mM), at 2 mM, pH = 5.0. **(E)** Mean Fourier transform infrared spectra of a) VG-6 and b) AG-9 using the KBR pellet method. Curve-fitting analysis performed on amide I bands of **(F)** VG-6 and **(G)** AG-9 to estimate the area of each component representing the secondary peptide structures. The second-derivative procedure was used to determine the position of the main underlying secondary structure components. Measured amide I spectra (solid curve) and theoretical spectra (point line).

In both media, the Hα and Cα CSDs of VG-6 and AG-9 were in agreement with the presence of preponderant random coil or β-turn conformations, excluding significant amounts of α-helix and β-sheet secondary structures (Tables 1, 2, 3 and 4) [34]. The ^3^J_NH–Hα_ coupling constant values of both elastin-like peptides were between 5.63 and 8.83 Hz, which also suggested the probable existence of random coil and β-turn structures in aqueous and micellar environments (Figure 3A–3D and Tables 1, 2, 3 and 4). The analysis of the temperature coefficient values between -4.9 and -9.6 ppb/K showed that all amide protons of VG-6 and AG-9 were unshielded from the solvent in both media, which was again in agreement with the presence of disordered conformations (Tables 1, 2, 3 and 4). All ROE patterns display some small and medium sequential d_Hα-NH_ and d_NH-NH_ ROEs consistent with random coil, β-sheet and β-turn secondary structures. However, the CSDs, ^3^J_NH–Hα_ coupling constants and temperature coefficient values excluded β-sheet structures in both media.

**Table 1:** Assignments of ^1^H and ^13^C resonances, chemical shift deviations (CSDs), ^3^J_NH-Hɑ_ coupling constants, and amide proton temperature coefficients of VG-6 in H_2_O/D_2_O (9:1 v/v), at 278 K

	Chemical shift (ppm)								
Residue	NH	H_ɑ_/C_ɑ_	H_β_/C_β_	H_γ_/C_γ_	H_δ_/C_δ_	^3^J_NH-Hɑ_ (Hz)	^1^H CSDs (ppm)	^13^C CSDs (ppm)	-∆δ/∆T (ppb/K)
V^1^	-	3.84/61.30	2.22/32.56	1.04/19.46, 20.14	-	-	−0.11	−1.55	-
G^2^	8.84	4.01, 4.07/44.48	-	-	-	6.07	0.04, 0.10	−0.17	6.4
V^3^	8.39	4.13/61.78	2.05/32.75	0.93, 0.94/20.05, 20.83	-	7.88	0.18	−1.07	8.2
A^4^	8.62	4.62/50.35	1.38/17.89	-	-	5.66	0.27	−2.00	9.6
P^5^	-	4.43/63.06	1.99, 2.30/31.97	2.05/27.18	3.70, 3.84/50.44	-	−0.01	0.31	-
G^6^	8.17	3.77/45.76	-	-	-	5.90	−0.20	0.91	9.5

**Table 2:** Assignments of ^1^H and ^13^C resonances, chemical shift deviations (CSDs), ^3^J_NH-Hɑ_ coupling constants, and amide proton temperature coefficients of VG-6 in DPC-*d*_*38*_, at 278 K

	Chemical shift (ppm)								
Residue	NH	H_ɑ_/C_ɑ_	H_β_/C_β_	H_γ_/C_γ_	H_δ_/C_δ_	^3^J_NH-Hɑ_ (Hz)	^1^H CSDs (ppm)	^13^C CSDs (ppm)	-∆δ/∆T (ppb/K)
V^1^	-	3.80/60.88	2.18/32.04	1.00/19.07, 19.70	-	-	−0.15	−1.97	-
G^2^	8.81	3.97, 4.02/44.20	-	-	-	6.00	0.00, 0.05	−0.65	5.9
V^3^	8.35	4.08/61.35	2.01/32.28	0.89, 0.90/19.40, 20.40	-	7.86	0.23	−1.50	7.5
A^4^	8.57	4.57/49.86	1.34/17.42	-	-	5.65	0.27	−2.49	9.1
P^5^	-	4.39/62.60	1.94, 2.25/31.57	2.00/26.65	3.65, 3.79/49.94	-	−0.05	−0.15	-
G^6^	8.12	3.72/45.33	-	-	-	5.91	−0.25	0.48	8.9

**Table 3:** Assignments of ^1^H and ^13^C resonances, chemical shift deviations (CSDs), ^3^J_NH-Hɑ_ coupling constants, and amide proton temperature coefficients of AG-9 in H_2_O/D_2_O (9:1 v/v), at 278 K

	Chemical shift (ppm)								
Residue	NH	H_ɑ_/C_ɑ_	H_β_/C_β_	H_γ_/C_γ_	H_δ_/C_δ_	^3^J_NH-Ha_ (Hz)	^1^H CSDs (ppm)	^13^C CSDs (ppm)	-∆δ/∆T (ppb/K)
A^1^	-	4.13/51.89	1.54/19.34	-	-	-	−0.22	−0.46	-
G^2^	8.74	3.97, 4.05/44.79	-	-	-	6.08	0.00, 0.08	−0.06	5.9
V^3^	8.41	4.43/60.18	2.09/32.65	0.95, 0.99/20.32, 20.91	-	7.72	0.48	−2.67	9.4
P^4^	-	4.41/63.63	1.93, 2.31/32.26	2.00, 2.07/27.52	3.71, 3.93/51.1	-	−0.03	0.88	-
G^5^	8.63	3.95/45.18	-	-	-	6.04	−0.02	0.33	6.7
L^6^	8.27	4.38/55.34	1.64/42.42	1.64/26.93	0.88, 0.93/23.48, 24.86-	6.60	0.21	−0.21	6.6
G^7^	8.66	3.96/45.38	-	-	-	6.10	−0.01	0.53	6.3
V^8^	8.09	4.22/62.04	2.15/32.95	0.90, 0.94/20.03, 21.21	-	8.61	0.27	−0.81	6.4
G^9^	8.28	3.73, 3.80/46.07	-	-	-	5.63	-0.24, -0.17	1.22	7.3

**Table 4:** Assignments of ^1^H and ^13^C resonances, chemical shift deviations (CSD), ^3^J_NH-Hɑ_ coupling constants, and amide proton temperature coefficients of AG-9 in DPC-*d*_*38*_, at 278 K

	Chemical shift (ppm)								
Residue	NH	H_ɑ_/C_ɑ_	H_β_/C_β_	H_γ_/C_γ_	H_δ_/C_δ_	^3^J_NH-Ha_ (Hz)	^1^H CSDs (ppm)	^13^C CSDs (ppm)	-∆δ/∆T (ppb/K)
A^1^	-	4.09/51.19	1.50/18.68	-	-	-	−0.26	−1.16	-
G^2^	8.71	3.92, 4.00/44.08	-	-	-	6.09	−0.05, 0.03	−0.77	4.9
V^3^	8.38	4.37/59.48	2.04/32.04	0.92, 0.95/19.77, 20.32	-	7.88	0.42	-3.37	8.4
P^4^	-	4.36/63.00	1.88, 2.27/31.54	1.95, 2.03/26.88	3.69, 3.89/50.44	-	−0.08	0.25	-
G^5^	8.57	3.90/44.55	-	-	-	6.00	−0.07	−0.30	6.1
L^6^	8.22	4.34/54.55	1.63/41.81	1.63/26.34	0.84, 0.89/23.00, 24.31	7.32	0.17	−1.00	5.9
G^7^	8.60	3.92/44.73	-	-	-	6.09	−0.05	−0.12	5.7
V^8^	8.04	4.18/61.35	2.10/32.36	0.86, 0.90/19.30, 20.55	-	8.83	0.23	−1.50	5.1
G^9^	8.25	3.68, 3.76/45.33	-	-	-	5.97	−0.29, −0.21	0.48	6.6

All NMR structural parameters led us to assume that VG-6 and AG-9 probably present a mixture of random coil and β-turn structures in aqueous and micellar media. This latter conclusion is in agreement with our previous CD results and with many elastin-like peptide structure studies described in the literature [35].

### FTIR spectroscopy

Figure 3E shows the mean FTIR spectra of VG-6 and AG-9. These spectra are dominated by two absorbance bands at 1666 and 1542 cm^−1^ known as the amide I and II bands, respectively. The amide I vibration, absorbing near 1666 cm^−1^, arises mainly from the C=O stretching vibration of the peptide bond. Comparison between these spectra shows that the most significant difference was located in the 1600-1700 cm^-1^ spectral region. In fact, the amide I band was located at 1670 cm^-1^ for VG-6 but shifted to 1664 cm^-1^ for the AG-9 peptide. This difference was associated with changes in peptide secondary structure. The amide II mode was attributed to the bending mode of the NH and the CN stretching vibration. The frequency of this band was the same between the spectra of these two peptides. The second-order derivative was applied to the amide I band to identify the peak frequencies of α-helix, β-sheet, β-turn and random (or unordered) structures. A curve fitting procedure was used to calculate the peak areas of the underlying bands to estimate the contribution of a definite type of secondary structure. This approach revealed that the amide I band consisted of bands at 1695, 1676, 1658, 1636, and 1617 cm^-1^ (Figure 3F–3G). A random coil structure was generally assigned to the band at 1658 cm^-1^ [36]. A band at 1676 cm^-1^ was commonly observed for antiparallel β-turns [37]. The bands at 1617, 1636, and 1695 cm^-1^ were assigned to antiparallel β-sheet conformations [36, 38]. Comparison between the secondary structures of these peptides showed a significant difference in the random coil and β-sheet structures (Table 5). The β-sheet structure at 1617 was absent in the VG-6 spectrum but appeared in the AG-9 conformation. The high random coil content in the AG-9 peptide could be due to its large number of glycine residues relative to VG-6.

**Table 5:** Assignment of amide I band positions and estimation the area of each component representing secondary structures of VG-6 and AG-9 peptides

Frequency	Secondary structure	% Area	
		VG-6	AG-9
**1617**	β-sheet		5.8
**1633-1636**	Antiparallel β-sheet	36.4	24.2
**1656-1658**	Random coil	18.2	27.7
**1675-1676**	β-turn	29.9	26.0
**1694-1695**	β-sheet	15.5	16.3

### Supramolecular organization

**Figure 4:**
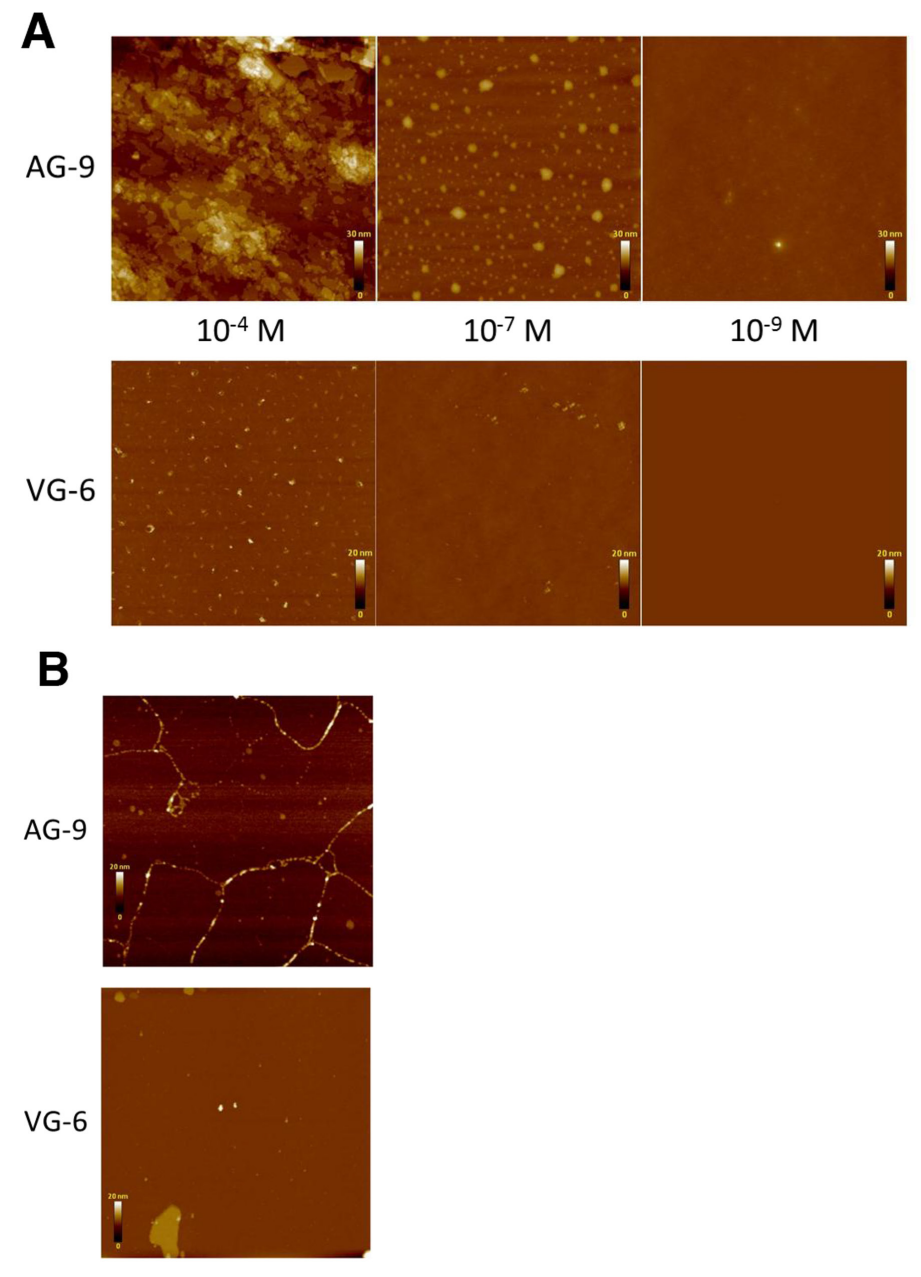
Analysis of supramolecular organization by atomic force microscopy (AFM) of VG-6 and AG-9 Observations were performed on peptides dissolved in water at 10^-4^, 10^-7^ and 10^-9^ M. **(A)** AFM tapping mode images of VG-6 and AG-9 on silicium in air. **(B)** AFM tapping mode images of VG-6 and AG-9 at 10^-4^ M on mica in liquid.

The supramolecular organizations of VG-6 and AG-9 spread on mica and silicium were investigated by AFM in different environmental conditions, i.e., in air or liquid and at different concentrations. Preliminary experiments were carried out in air. At 10^-4^ M, AFM tapping mode images of AG-9 exhibited a sheet-like assembly of plates ranging from 10 nm to 1 μm in width (Figure 4A). At 10^-7^ M, the peptides gathered in spots of variable size (from a few nm up to 100 nm) but constant height (approximately 25 nm). At lower concentrations, the spots were much more scattered and harder to find. At 10^-4^ M, VG-6 showed dot-like structures of a few nm (up to tens of nm) in width and 20 nm in height. At lower concentrations, the dots were much more spread and difficult to locate. This prompted us to test both peptides at the 10^-4^ M concentration in liquid. Interestingly, AG-9 did not show any plate-like structures as in air but rather existed as fibrils of 5 to 20 nm in height and up to several tens of nm in length, possibly revealing the presence of subunits or various degrees of peptide assemblies (Figure 4B). Interestingly, some raft-like structures of constant height (10 nm) and average size of (10 to 30 nm) were simultaneously observed. Under the same conditions, VG-6 did not seem to exhibit fibrils but rather existed as raft-like plates of 10 nm in height and variable size (from 100 nm up to 1 μm) and sporadic spots.

### The AG-9 peptide induces *in vivo* tumor growth

**Figure 5:**
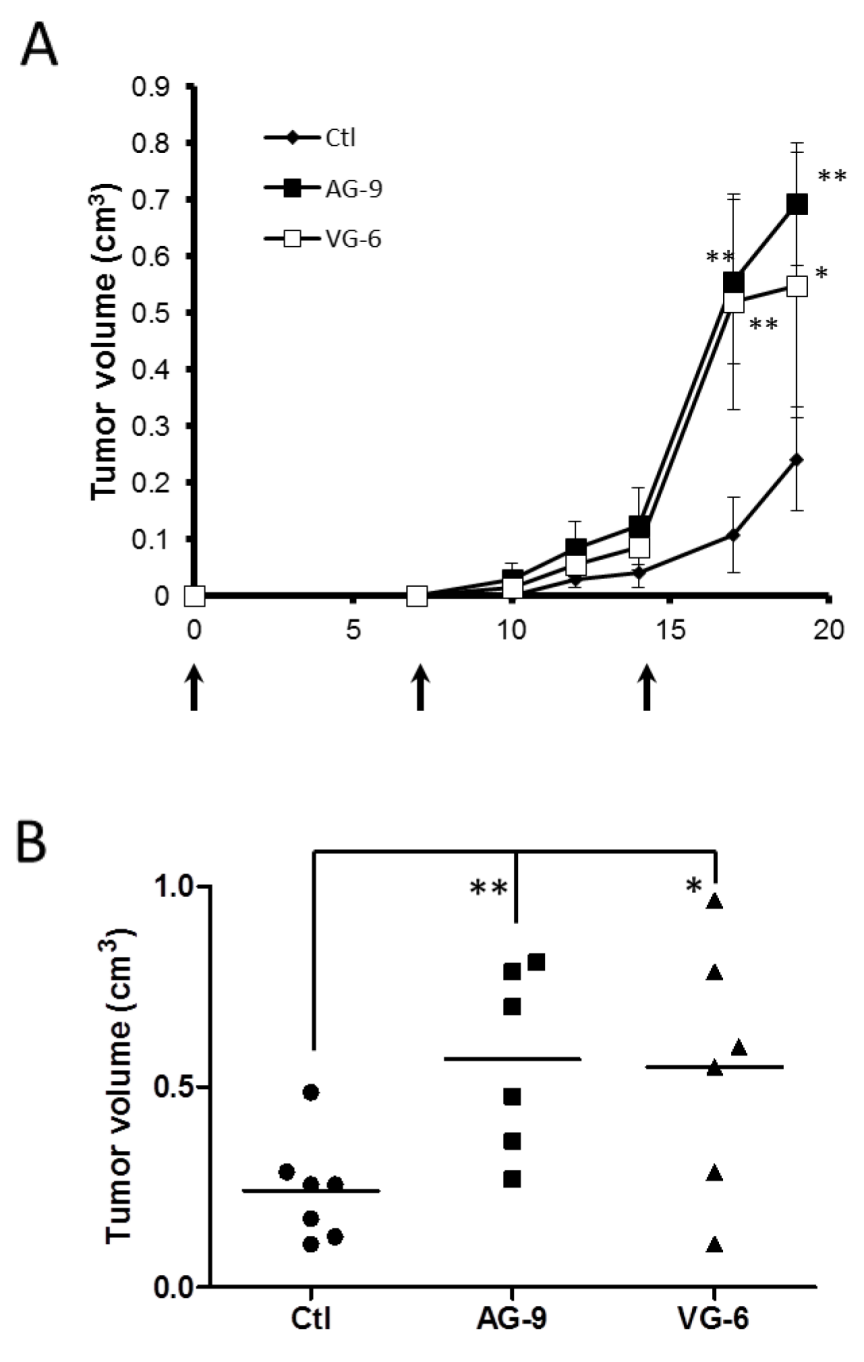
AG-9 induces *in vivo* tumor growth **(A)** Kinetics of tumor growth assessed by Vernier caliper measurements in control (♦, n=7), VG-6 (□, n=6) and AG-9 (■, n=6) mice. **(B)** tumor volume of B16-F1 tumors from control (●, n=7), VG-6 (▲, n=6) and AG-9 (■, n=6) mice 19 days after tumor implantation. Data are means ± SEM. ^*^P <0.05; ^**^P <0.01.

To determine the effects of VG-6 and AG-9 on cancer development, control B16-F1 cells or B16-F1 preincubated in the presence of VG-6 and AG-9 peptides (10^-4^M) were subcutaneously injected into the right sides of syngenic C57Bl6 mice. Control and VG-6- and AG-9-treated mice were killed on day 19. Tumors were excised and measured. The mean tumor volume was increased by at least twofold in VG-6-treated mice vs control (2.27-fold; P<0.05) and AG-9-treated mice vs control (2.35-fold; P<0.01) (Figure 5A-5B). These results showed that AG-9 strongly enhanced tumor growth *in vivo* to a greater extent than did the VG-6 peptide.

### The AG-9 peptide induces *in vitro* cell growth, proteinase secretion, cell migration and cell adhesion

**Figure 6:**
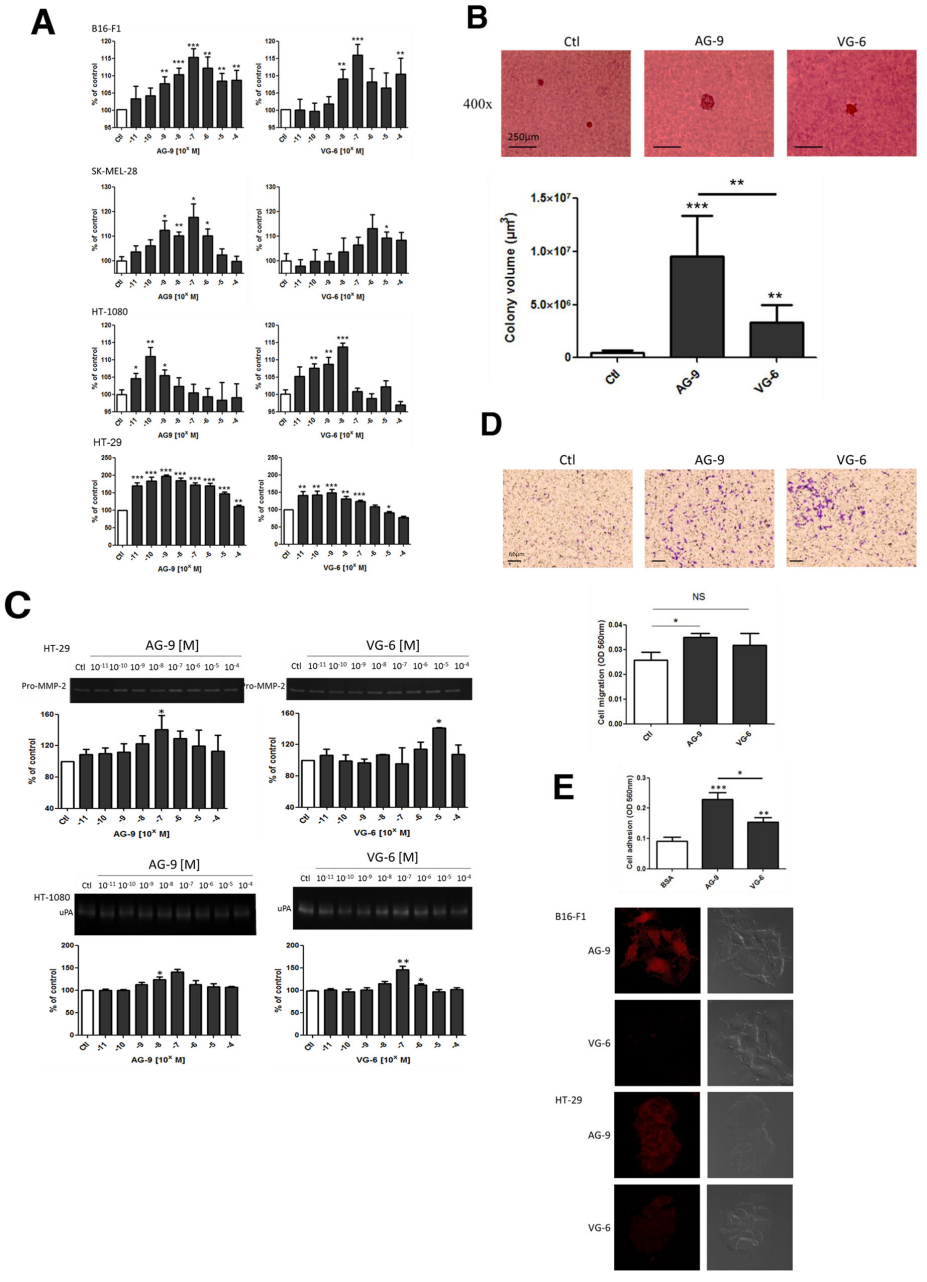
AG-9 induces *in vitro* cell growth, proteinase secretion and cell migration **(A)** AG-9-stimulated B16-F1, SK-MEL-28, HT-1080 and HT-29 cell proliferation. Significantly different from control: ^*^ P<0.05; ^**^ P<0.01; ^***^ P<0.001. **(B)** Colony formation in soft agar was measured after 14 days of incubation. ^**^ P<0.01; ^***^ P<0.001. **(C)** Zymography quantification of MMP-2 secretion by HT-29 and HT-1080 cells in the presence of AG-9 and VG-6 (10^-11^ to 10^-4^M). **(D)** AG-9 increases HT-29 cell migration. Representative photomicrographs and quantification of HT-29 migration induced by AG-9 and VG-6 (10^-7^ M) after 24 h of incubation. The histogram represents the mean ± SEM of 4 replicates. ^*^Significantly different from control (NS: not significant; ^*^ P<0.05). **(E)** Effect of AG-9 and VG-6 on HT-1080 cell adhesion and binding of AG-9-T and VG-6-T to B16-F1 and SK-MEL-28 cells. Data are the means ± SEM. ^**^ P <0.01; ^***^ P <0.001.

To confirm that *in vivo* tumor growth is directly mediated by VG-6 and AG-9, that is, that EDPs do not signal *via* other ECM components, we evaluated their pro-proliferative abilities in B16-F1, SK-MEL-28, HT-1080 and HT-29 cells. The stimulation of cancer cells with VG-6 and AG-9 in the presence of 2.5% FBS triggered cell proliferation at 48 h in a dose-dependent manner (Figure 6A). AG-9 stimulated cell proliferation at a lower concentration than did VG-6. The optimal AG-9 concentration was 10^-10^ to 10^-7^ M, whereas the optimal VG-6 concentration was 10^-8^ to 10^-6^ M.

To measure the effect of VG-6 and AG-9 on proliferation in a semi-solid culture medium, the anchorage-independent growth of cancer cells in soft agar was investigated. After 14 days of cell culture, the volumes of cancer cell colonies were increased in the presence of AG-9 and VG-6 by 2043±33% and 650±25%, respectively (Figure 6B). These results consequently demonstrate that VG-6 and AG-9 directly enhanced cancer growth.

Owing to the important role of proteinases such as MMP-2 and uPa in the cancer invasion process, we investigated their expression by cancer cells in the presence of VG-6 or AG-9. These analyses demonstrated increased secretion of both proteinases by HT-29 and HT-1080 cells in a dose-dependent manner (Figure 6C). Less mobile than HT-1080 cancer cells, HT-29 cells were tested for their ability to migrate following VG-6 or AG-9 stimulation in an *in vitro* system. These experiments were performed using FBS-free conditions to evade cell proliferation. We found that AG-9 significantly stimulated more HT-29 cell migration then did VG-6 (p<0.05) (Figure 6D). In parallel, the abilities of cancer cells to bind VG-6 and AG-9 were tested and compared to BSA adhesion. We found that cancer cell adhesion to VG-6 was significantly higher than that to BSA (P<0.01) and adhesion to AG-9 was more than twofold higher than BSA adhesion (P<0.001) and 1.5-fold higher to VG-6 (Figure 6E). These results were confirmed by confocal microscopy analysis of AG-9 and VG-6 binding to B16-F1 and SK-MEL-28 cells.

### The AG-9 peptide stimulates angiogensesis

**Figure 7:**
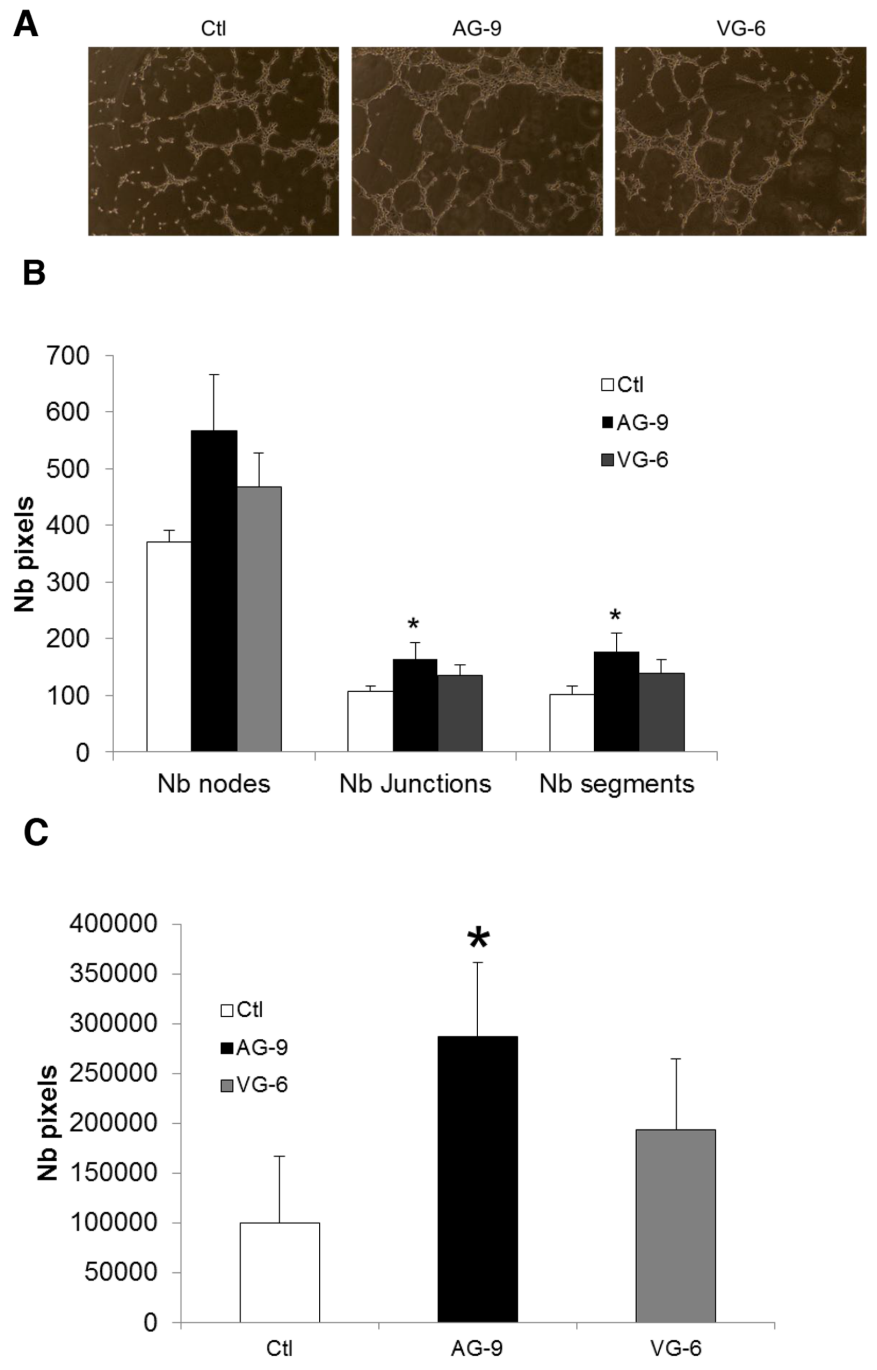
AG-9 induces HUVEC pseudotube formation **(A)** Representative photomicrographs of pseudotube formation by HUVECs incubated with or without 10^-7^ M of AG-9 or VG-6. **(B)** Quantification of the number of segments, the number of nodes and the total pseudotube length. **(C)** Quantification of the total pseudotube area. The values are the means ± SD of 8 replicates. ^*^Significantly different from control (^*^P<0.05).

Previous studies have shown that kappa-elastin and VG-6 accelerate angiogenesis in the chick chorio-allantoic membrane in an *in vivo* model [15]. To study the effect of AG-9 on angiogenesis, pseudotube formation from HUVECs in matrigel was analyzed after 8 h of incubation. We found that compared with control and VG-6 conditions, AG-9 at a concentration of 10^-7^ M stimulated angiogenesis by increasing the numbers of nodes, junctions and segments (Figure 7A–7B). In the presence of AG-9, pseudotube network formation was 2.88-fold greater under AG-9 conditions than in the control and 1.48-fold higher under AG-9 conditions than under VG-6 conditions (Figure 7C).

## DISCUSSION

Within the past thirty years, many articles describing the important roles of different EDPs have been published. Among the EDPs, VG-6 is the most frequently described because this peptide modulates a large number of biological activities and is associated with physiologic and pathologic processes [8, 11, 15, 21, 39–42]. We previously demonstrated that diverse peptides containing GxxPG sequences can induce similar cellular effects as a “principal” VGVAPG ligand of the ERC [39]. Our biophysical studies suggested that this peculiar consensus sequence stabilizes a type VIII β-turn in several similar, but not identical, peptides to maintain a sufficient conformation to be recognized by the elastin receptor complex at the fibroblast cell surface.

One aim of this study was the characterization of AG-9 and comparison of these data to those of the well-known VG-6 reference peptide. Unlike VG-6, the results demonstrated that AG-9 self-assembles and that during the aggregation process, it adopts a β-conformation. NMR data recorded in an aqueous solution and in agreement with CD results showed that in AG-9, β-strands coexist with random coil conformations. Moreover, from FTIR spectra, the position of the dominant peak observed at 1626 cm^−1^ was close to that observed for (VGGVG)_3_ and poly(VGGVG) fibrils at 1617–1635 cm^− 1^, which is also indicative of cross-β-sheet structures [43, 44]. Our data showed relevant changes in FTIR spectra as a result of conformational changes between the VG-6 and AG-9 peptides. The amide I band is more sensitive to the protein secondary structural components than is its amide II counterpart. Even with small peptides, FTIR results are complimentary to the information obtained from CD spectroscopy. Small peptides are structurally useful (regardless of their size and amino acid composition) and can be easily characterized by several spectroscopic methods. A previous study investigated two elastin-like poly(pentapeptides), poly(AV_1_GV_2_P) and poly(G_1_V_1_G_2_V_2_P), in water and in a solid state by attenuated total reflectance (ATR) FTIR [45]. This study demonstrated that the amide groups mutually interact to form a β-sheet structure, stabilized mostly in a planar form by hydration. Different techniques such as differential scanning calorimetry (DSC), CD, UV absorption, FTIR and NMR spectroscopy have been used to study the secondary structures of C(GVGVP)_6_ [46]. These results suggested that the antiparallel β-sheet forms the basis of the conformation.

With this study, we highlighted that xGxPGxGxG peptides such as AG-9 can modulate the same biological activities as xGxxPG in a more effective manner. AG-9 promotes greater tumor progression *in vitro* and *in vivo*. Cell proliferation, cell adhesion, cell migration, proteinase secretion and angiogenesis are systematically modulated by AG-9 in a manner superior to that shown by VG-6. Moreover, we found that all of these effects are regulated at low AG-9 peptide concentrations of approximately 10^-7^ M, whereas the optimal VG-6 concentration is approximately 10^-5^ M. In view of these results, the xGxPGxGxG receptor identification is essential. Maeda I *et al.* showed that elastin-derived nonapeptides are crucial for inducing macrophage migration involving a receptor insensitive to lactose and excluding the elastin receptor complex [19]. Our previous results confirmed the presence of a nonapeptide-receptor different from the ERC, αVβ3 integrin and galectin-3 [20]. Protease secretion by lung carcinoma cell lines could not be inhibited by lactose and V14, two ERC antagonists, or by blocking antibodies against αVβ3 integrin and galectin-3. Given these data, the implication of a single elastin receptor in tumor progression is no longer possible [47]. It is therefore important to consider the existence of a currently unknown or forgotten elastin receptor [39]. In the future, the measurement of AG-9 in patient sera and the detection of the AG-9 receptor in cancer patients could improve the diagnostics or prognostics and will constitute innovative approaches to allow for major clinical developments. Given its properties such as better adhesion to the tumor cell surface and aggregation capacities, ELPs derived from xGxPGxGxG should be tested in antitumor therapies but also biomaterials for tissue engineering.

## MATERIALS AND METHODS

### Comparative analyses of ELN-containing sequences

A list of sequences used in this study is provided in Supplementary Table 1. The multi-species peptide sequences were from Ensembl (http://www.ensembl.org). Alignment of peptide sequences was performed using the Kalign server (http://msa.sbc.su.se/cgi-bin/msa.cgi). The comparisons were computed as local alignments using the default parameters of the program. Phylogenetic tree comparisons were made using the global alignment program Kalign, which is available at EBI (http://www.ebi.ac.uk), to calculate the identity and similarity.

### Reagents

VG-6, AG-9, TAMRA-AG-9 and TAMRA-VG-6 peptides were purchased from Proteogenix (Schiltigheim, France). D_2_O and dodecylphosphorylcholine-*d*_*38*_ (DPC-*d*_*38*_) detergents were purchased from Eurisotop (Gif-sur-Yvette, France). Sodium 2,2-dimethyl-2-silapentane-*d*_*6*_-5-sulfonate (DSS) and 2,2,2-trifluoroethanol (TFE) were purchased from Sigma-Aldrich (St. Quentin Fallavier, France).

### Cell culture

Human colon adenocarcinoma (HT-29), fibrosarcoma cells (HT-1080), human and mouse melanoma cells (SK-MEL-28 and B16-F1, respectively) and human umbilical vein endothelial cells (HUVECs) were obtained from the American Type Culture Collection (Rockville, MD). HT-29 cells were cultured in McCoy’s 5A supplemented with 5% Fetal Bovine Serum (FBS) and HT-1080, SK-MEL-28 and B16-F1 cells were cultured in Dulbecco’s modified Eagle’s medium (DMEM) (Gibco-BRL) supplemented with 10% FBS.

HUVECs were grown in endothelial cell growth medium (ECGM, PromoCell, France) supplemented with 2% FBS and growth factors according to the manufacturer’s instructions in Nunclon® flasks (Dutscher Brumath, France) at +37°C in a humid atmosphere (5% CO_2_, 95% air). For all experiments, a PromoCell DetachKit was used for the detachment of HUVECs according to the manufacturer’s instructions.

### Animals

C57Bl6 mice (average body weight, 16–18 g) were purchased from Harlan France (Gannat, France). Animals were individually caged and given food and water *ad libitum*. They were kept in a room with constant temperature and humidity. All mice were acclimatized to our laboratory conditions for 1 week before starting the experiments. The *in vivo* experiments were conducted according to the ethical guidelines of the Centre National de la Recherche Scientifique.

### *In vivo* tumor growth measurement

A suspension of B16-F1 cells (2.5 × 10^5^ in 0.1 mL DMEM) was subcutaneously injected into the left sides of syngeneic C57Bl6 mice. B16-F1 cells were preincubated for 30 min with either DMEM (control), AG-9 (10^-4^M), or the reference peptide, VG-6, at the same concentration before the initial injection. Control medium, AG-9 (10 mg/kg mouse weight), and VG-6 (10 mg/kg mouse weight) intraperitoneal injections were performed at days 7 and 14. Each group included six or seven mice. Tumor volumes were determined according to V = 1/2 A × B^2^, where A denotes the largest dimension of the tumor and B represents the smallest dimension [48]. Tumor volume was measured on days 7, 10, 12, 14 and 17. At day 17, the mice were sacrificed, and tumors were surgically excised for morphologic and biochemical studies.

### Cell proliferation

To measure cell proliferation, 2,000 cells were plated into 96-well plates and incubated for 48 h with VG-6 and AG-9 in culture media supplemented with 2.5% FBS. After cell fixation with glutaraldehyde, cell proliferation was measured using a crystal violet colorimetric assay [12].

### 3D Anchorage-independent growth

Soft agar assays were carried out in 6-well plates. Each well contained the following layers: bottom layer: 1 mL of 0.6% agar; middle layer: 0.3% agar with HT-29 cell suspension (5.x10^3^ cells/well); and top layer: 1 mL of 0.6% agar. The agar was mixed 1:1 with 2× RPMI medium. Each layer contained 10^-7^ M VG-6 or AG-9 or no peptide. Cell colonies were photographed after 14 days of incubation, and their volumes were estimated using ImageJ software.

### Migration assay

Migration tests were performed in 8-μm pore size polyethylene terephthalate membrane cell culture inserts (BD FALCON™ Cell Culture Inserts, BD Biosciences). The upper compartment was seeded with 5x10^4^ cells in FCS-free growth medium with or without 10^-7^ M synthetic elastin peptides for 24, 48 or 72 h at +37°C. The lower compartment was also filled with FCS-free growth medium. Thus, migration assays were performed without the addition of a chemoattractant. After incubation, the cells were washed with phosphate-buffered saline (PBS), fixed with methanol and stained with crystal violet for 15 min. The remaining cells were removed from the upper side of the membrane by scrubbing. Crystal violet staining of the migrated cells on the lower side was eluted in 10% acetic acid, and the absorbance was read at 560 nm.

### Adhesion assay

For cell adhesion assays, 96-well microtiter plates were purchased from Dutscher™, (Brumath, France). The plates were coated with the different substrates described below diluted in carbonate buffer (0.2 M sodium carbonate, 0.2 M sodium bicarbonate, pH 9.6) overnight at +4°C. Bovine serum albumin (BSA) (Euromedex™, Souffelweyersheim, France) at 1 μg/well was used as a negative control. AG-9 and VG-6 peptides were coated in the same carbonate buffer at 1 μg/well. Cells were detached with a versene buffer (126 mM NaCl, 5 mM KCl, 1 mM EDTA, and 50 mM HEPES) and centrifuged at 300 × g for 5 min at room temperature. The cells were then incubated with 2 mM CaCl_2_ and 0.5 mM MgCl_2_ in DMEM. After being counted, the cells were seeded at a density of 30,000 cells in 100 μL/well and incubated for 2 h at +37°C in 5% CO_2_. Unattached cells were removed by washing with PBS. Attached cells were fixed with 1.1% glutaraldehyde in PBS for 20 min. After washing, the cells were stained with 0.1% crystal violet for 20 min. After extensive washing with distilled water and air-drying, crystal violet was eluted in 100 μL/well of 10% acetic acid. The absorbance was then measured at 560 nm.

### Preparation of conditioned media and zymography analysis

Cells were grown to subconfluence in 24-well culture plates (Nunc, distributed by Polylabo, Strasbourg, France) in 10% serum-containing medium. After 16 h of serum deprivation, synthetic peptides were added to serum-free culture medium, and the cells were incubated for 24 h. Conditioned media were harvested and centrifuged at 10,000 × g for 10 min at +4°C to remove cellular debris. Conditioned media samples from cancer cell cultures were analyzed by zymography electrophoresis as previously described [49].

### Confocal microscopy

Cells were plated on glass slides and incubated in 10% serum-containing medium for 16 h. Synthetic elastin peptides were then added to serum-free culture medium and cells were incubated for different times at +4°C and +37°C. After several washes, the cells were fixed for 5 min with 4% paraformaldehyde. The slides were washed with PBS-T and saturated in PBS-T with 5% BSA. The cells were then incubated for 1 h at room temperature with the first antibodies diluted 1/400 in PBS-T with 1% BSA. The slides were washed in PBS-T and cells were incubated for 30 min with the Alexa 488- or Alexa 568-conjugated secondary antibodies diluted 1/1000 in PBS-T with 1% BSA. Cells were then washed with PBS-T. Control preparations were incubated with omission of the first antibody. Immunofluorescence-labeled cell preparations were evaluated using a Zeiss LSM 710^®^ NLO confocal laser scanning microscope (Carl ZEISS SAS, Marly le roi, France) with a 63x oil-immersion objective (ON 1.4) coupled with a CHAMELEON femtosecond Titanium-Sapphire Laser (Coherent, Santa Clara, CA).

Alexa 488 and 568 were sequentially excited by the 488 nm line of an argon laser and a diode laser at 561 nm. Emitted signals were respectively collected with 493-560 nm and 570-700 bandpass filters. Image acquisitions were performed with ZEN Software (Carl ZEISS SAS, Marly le roi, France), and all acquisition settings were maintained constant between specimens.

### Capillary tube formation on matrigel

The ability of HUVECs to form capillary tube structures was investigated on a Matrigel matrix. A 48-well plates were coated with Matrigel (7 mg/mL), which was allowed to polymerize at +37°C for 2 hours. After 2 hours, 50,000 HUVECs were suspended in ECGM supplemented with growth factors (PromoCell, France) with or without 10^-7^ M AG-9 or VG-6 and seeded into each well. Plates were incubated at +37°C in a humid atmosphere (5% CO_2_, 95% air) for 8 hours. Capillary tube formation was imaged after 8 hours under an inverted light microscope. Quantitative evaluation of the capillary tubes was completed using the ImageJ software analysis program (NIH, Bethesda, USA). The number of nodes, the number of segments and the total length of the capillary tube structures were measured. The results are presented as mean ± SD, (n = 8).

### Circular dichroism

Circular dichroism (CD) spectra were recorded on a JASCO J-815 Spectropolarimeter (JASCO, Milan, Italy) under constant nitrogen flush over the wavelength range of 190 – 260 nm by using a 0.1-cm path-length quartz cell (internal volume 200 μL). Spectral measurements were carried out at 293 K, with a 20 nm/min scan speed and a bandwidth of 1 nm. VG-6 and AG-9 were separately dissolved at a concentration of 0.1 mM in Milli-Q water, in Milli-Q water/TFE (7:3 v/v), in Milli-Q water/methanol (7:3 v/v) and in 80 mM DPC-*d*_*38*_ detergents. CD spectra represented the average of 3 scans and all final spectra were obtained after subtracting the blank. Data are expressed in terms of [Θ] and the molar ellipticity in units of degree cm^2^.dmol^− 1^.

### NMR spectroscopy

VG-6 and AG-9 were respectively solubilized at concentrations of 5 and 7.5 mM in H_2_O/D_2_O (9:1 v/v), at pH = 5.0. Both peptides were also dissolved at a concentration of 2 mM in H_2_O/D_2_O (9:1 v/v), at pH = 5.0, to which DPC-*d*_*38*_ detergents were added at a concentration of 160 mM in order to ensure a ratio of at least 1 micelle per peptide (assuming an average aggregation number of ∼60 detergent molecules per micelle). DSS was used as an internal reference (0.1 mM) for ^1^H chemical shift calibration.

All NMR experiments were performed on a Bruker Avance AVIII-600 NMR spectrometer equipped with a 5-mm TCI cryoprobe using Bruker TOPSPIN Software (Rheinstetten, Germany). Static field gradient pulses were generated by a 10-A amplifier so that the sample was submitted to a nominal 0.613 Tm-1 gradient. Gradient pulses were followed by a 200-μs recovery delay. Temperature was controlled by a Bruker variable temperature (BSVT) unit supplied with chilled air produced by a Bruker cooling unit (BCU-Xtreme).

One-dimensional spectra were acquired in Fourier mode with quadrature detection, and the water signal was suppressed by double pulsed field gradient spin echo [50]. Two-dimensional TOtal Correlation SpectroscopY (TOCSY) and Rotating Frame Overhauser SpectroscopY (ROESY) spectra were recorded in the phase-sensitive mode using the States-TPPI method. Their spectra sizes were 1k*4k with 8 scans per FID and 1.5-s relaxation delay. The spinlock (MLEV-17) mixing time was 100 ms for the TOCSY experiments, whereas the mixing time of 150 ms was applied to the ROESY experiments. The data matrixes were multiplied in both dimensions by a shifted sine bell function (SSB = 2) before zero filling to a 1k * 4k size. Amide proton temperature coefficients were measured from 1D ^1^H NMR spectra recorded in 5-K increments from 278 K to 313 K. ^1^H sequential resonance assignments were accomplished according to the approach described by Wüthrich K [33].

### FTIR spectroscopy

Several Fourier transform infrared (FTIR) spectra were recorded on two solid VG-6 and AG-9 samples using the potassium bromide (KBR) pellet method. The pellets were prepared by mixing 1 mg of solid peptide samples with 99 mg of dried KBR powder. The mixtures were finely pulverized and placed into a pellet-forming system. A 10-ton pressure force was applied to the sample for 15 minutes under a vacuum to form thin transparent pellets. FTIR spectra were obtained using a Perkin Elmer spectrometer (spectrum Bx series) equipped with a KBr beam-splitter and deuterated triglycine sulfate (DTGS) detector. The spectra were recorded on samples in the spectral range of 600-4000 cm^-1^ in transmission mode by averaging 100 scans with a spectral resolution of 4 cm^-1^. A background spectrum was recorded from KBR pellets without peptide in the same experimental conditions. This spectrum was used for subtraction from the peptide spectra. To allow meaningful comparisons, all FT-IRM data were uniformly preprocessed. Data were cut to the fingerprint region (1300 to 2000) and smoothed using a seven-point Savitzky-Golay algorithm. The resulting spectra were then normalized using a standard normal variate (SNV) procedure [51].

Curve-fitting analysis was applied on amide I region data (1600–1700 cm^−1^) to obtain quantitative estimations of the secondary structures of VG-6 and AG-9. This estimation was based on the assumption that the amide I band of the peptide can be considered as a linear combination of α-helix, β-sheet, β-turn and random (or unordered) secondary features [52]. A second derivative procedure was applied to this band to identify the center position (peak frequencies) of the main components within the amide I profile. The band shape of each underlying band was considered as a Gaussian A curve-fitting procedure performed to estimate the area of each component representing the secondary structures of the peptides [53]. A least-square iterative analysis was then completed to obtain the best fit.

### Atomic force microscopy (AFM)

Ten microliters of each peptide solution (10^-4^, 10^-7^ and 10^-9^ M) was deposited on freshly cleaved mica disks. AFM observations were carried out using a Multimode 8 (Bruker, Billerica, USA) at room temperature, either in air (with RTESPA probes, having a nominal tip radius of less than 5 nm and a spring constant of 42 N/m) or in purified water (with DNP probes, having a nominal tip radius of 5 nm and a spring constant ranging from 0.24 to 0.32 N/m). In both cases, we used intermittent contact mode to reduce the shear and friction forces on the sample at scan rates less than 1.5 Hz. All probes were purchased from Bruker (Billerica, USA).

### Statistical analysis

For *in vitro* experiments, statistical analyses were performed using Student’s *t*-test, and the results are expressed as the mean ± SD. For *in vivo* experiments, volumes of primary tumors were statistically analyzed using the Mann and Whitney nonparametric *U* test. The results were considered significant when ^*^ P < 0.05, ^**^ P < 0.01, ^***^ P < 0.001.

## SUPPLEMENTARY MATERIALS TABLE




