# Supplementary material for: Structural characterization and in vivo pro-tumor properties of a highly conserved matrikine
Source: Oncotarget. 2018 Apr 3;9(25):17839–57. doi: 10.18632/oncotarget.24894 (PMC5915158; doi:10.18632/oncotarget.24894)
Supplement: Supplementary file 1 [file oncotarget-09-17839-s001.pdf]

# Structural characterization and *in vivo* pro-tumor properties of a highly conserved matrikine

## SUPPLEMENTARY MATERIALS

**Supplementary Table 1: ELN-containing sequences used for comparative analyses.**

**See Supplementary File 1**
