# Supplementary material for: Structural characterization and in vivo pro-tumor properties of a highly conserved matrikine
Source: Oncotarget. 2018 Apr 3;9(25):17839–57. doi: 10.18632/oncotarget.24894 (PMC5915158; doi:10.18632/oncotarget.24894)
Supplement: Supplementary file 2 [file oncotarget-09-17839-s002.docx]

**Supplementary Table 1: ELN-containing sequences used for comparative analyses.**
